# Temporal and Workshop Heterogeneity of Microbial Communities with Physicochemical Properties and Flavor Substances During Stacked Fermentation of Sauce-Flavor Baijiu

**DOI:** 10.3390/foods14060924

**Published:** 2025-03-08

**Authors:** Jiao Niu, Yahan Yan, Guihu Zhang, Yi Shen, Wei Cheng, Hehe Li, Zhongfu Duan, Jinyuan Sun, Bowen Wang, Jihong Wu, Baoguo Sun

**Affiliations:** 1 Key Laboratory of Geriatric Nutrition and Health, Beijing Technology and Business University, Ministry of Education, Beijing 100048, China; niujiao.2009@163.com (J.N.); han15082969822@163.com (Y.Y.); zhanggh0634@163.com (G.Z.); sunjinyuan@btbu.edu.cn (J.S.); wangbw@btbu.edu.cn (B.W.); wujihong12@126.com (J.W.); sunbg@btbu.edu.cn (B.S.); 2 Sichuan Lang Jiu Co., Ltd., Luzhou 646699, China; shenyi@langjiu.cn (Y.S.); chengwei@langjiu.cn (W.C.); duanzfbtbu@126.com (Z.D.); 3 Beijing Key Laboratory of Quality and Safety, Beijing Technology and Business University, Beijing 100048, China

**Keywords:** sauce-flavor Baijiu, stacked fermentation, microbial community, physicochemical factors

## Abstract

Even within the same round of stacked fermentation, variations among production workshops can significantly influence microbial communities and physicochemical parameters. In this study, stacked fermented grains from the fourth round of sauce-flavor Baijiu production were utilized to explore the impact of spatial variations on fermentation. High-throughput sequencing technology was employed to comprehensively analyze the microbial community composition and its dynamic changes during the fourth cycle of stacked fermentation in sauce-flavor Baijiu across different workshops. Our results revealed that the predominant genera in both workshops included *Saccharomycetales*, *Thermomyces*, *Monascus*, *Ascomycota*, *Kroppenstedtia*, *Bacillus*, and *Virgibacillus*. Differences in physicochemical factors during the fermentation process led to distinct microbial successions among workshops. Key drivers of dominant microbial community succession, such as glucose, starch, ethanol, and temperature, were identified during the fourth round of stacked fermentation. Differences in the types and contents of significant flavor substances in different workshops are related to the complex role of the microbial community. Acetic acid is the most different flavor substance in both workshops, and the content of acetic acid affects the synthesis of ethyl ester substances, which has significant correlation with the regulation of fungal communities, especially *Saccharomycetales.* These findings provide valuable insights into the brewing mechanisms of the stacked fermentation process and offer guidance for formulating more refined control strategies to optimize the liquor-making process.

## 1. Introduction

Sauce-flavor Baijiu, a principal aroma category in Chinese Baijiu, is renowned for its complex and refined sauce-like fragrance and its enduring aroma that lingers in an empty glass [1]. The distinctive aromatic profiles of sauce-flavor Baijiu are intricately linked to its unique and elaborate production process as follows: First, fermented grains are mixed with Daqu and water. Subsequently, they are piled on the floor of the fermentation workshop for 2 to 11 days for stacking fermentation, the duration of which is contingent upon the ambient temperature. After that, the mixture is transferred to a cellar pool sealed with cellar mud and left for another month to undergo anaerobic fermentation. Next, the fermented grains retrieved from the mud-sealed cellar are distilled, and finally, the base Baijiu is collected. This process involves eight fermentation rounds to produce the desired base Baijiu [2]. Each fermentation round is divided into two main stages: stacking fermentation and cellar fermentation. Stacking fermentation is conducted in an open production workshop [3,4]. This process not only enriches the environmental microbiota, but also generates substrates and flavor precursors essential for subsequent cellar fermentation [5]. Owing to the open production environment, fluctuations in moisture, acidity, and temperature within the fermented grains have an impact on the microbial community structure. These changes are conducive to the growth and reproduction of microbes, playing a pivotal role in alcohol fermentation and the formation of aromatic substances during the cellar fermentation process [6]. Therefore, the dynamics of microbiota structure and physicochemical factors during the stacked fermentation need to be investigated to elucidate the fermentation mechanisms and flavor formation in sauce-flavor Baijiu.

Previous research has focused on the various stages of sauce-flavor Baijiu fermentation process [7,8]. Studies have demonstrated that during the third to fifth rounds of stacked fermentation, the predominant bacterial phylum is *Firmicutes*, while the dominant fungal phylum is *Ascomycetes*. Additionally, significant bacterial genera such as *Propionibacterium* and *Staphylococcus* were observed to negatively regulate the fermentation system [9]. Similar findings were observed in the second round of fermentation [10]. Moreover, comparative analysis of microbiota structures during the sixth round of stacked fermentation in old and new workshops revealed similar predominant genera, primarily, *Bacillus* spp. and *Aspergillus* spp. [11]. Variations in endogenous factors such as moisture, ethanol, and acidity were observed to influence microbial community, during microbial succession at different fermentation stages [7]. These studies provide a foundation for rationally controlling Baijiu production and improving both craft and quality standards for sauce-flavor Baijiu.

Flavor analyses have unveiled significant disparities in the quality of sauce-flavor Baijiu across different production rounds. The first and last two rounds of Baijiu are traditionally characterized by astringent flavors and are considered of lower quality. In contrast, the third, fourth, and fifth rounds, collectively referred to as “dahui Baijiu”, are renowned for their superior quality [12]. These rounds represent the core stages of the sauce-flavor Baijiu brewing process [13], with the fourth fermentation round producing the highest quantity and quality of Baijiu [14]. A systematic and comprehensive understanding of the microbial community structure during this stage is crucial for optimizing the fermentation process and achieving consistent high-quality sauce-flavor Baijiu.

The open and complex ecological environment of Baijiu brewing results in changes in microbiota in different workshops, leading to the varied metabolic synthesis of flavor substances and the diverged base Baijiu qualities. During the natural fermentation process, the structure of the microbial community is typically changed in response to the fermentation environment. It is influenced by factors such as temperature, humidity, the acidity of fermented grains, and fermentation substrates [2]. The structure of the microbial community and the profile of its metabolites can be significantly impacted by diverse environmental conditions. For instance, the distribution and diversity of Streptomyces are shaped by geographical and climatic factors. Geosmin is produced by this bacterium, which imparts earthy odors [15]. Even within the same geographical area, differences in Baijiu quality can be led to by variations in workshop conditions, mainly due to disparities in microbial ecology and physicochemical factors.

Stable liquor quality is regarded as the lifeblood of Baijiu enterprises, the driving force for their sustainable development, and the key to building a strong brand. The stacked fermentation mechanism of the fourth round of fermentation in the sauce-flavor Baijiu factory located at Erlang Town (Luzhou City, Sichuan Province, China) needs to be explored. This exploration is of great significance for enhancing Baijiu quality and stability and eliminating differences among different workshops.

In this study, the fermented grains from the fourth round stacking fermentation in different workshops of the sauce-flavor Baijiu factory were taken as the research objects. A correlation analysis was carried out between the microbial community structure and physicochemical components of the fermented grains. Meanwhile, the flavor differences in the fermented grains were analyzed, with the aim of providing a theoretical basis for the stacking fermentation mechanism of the fourth round and the control of the fermentation process.

## 2. Materials and Methods

### 2.1. Sample Collection

Samples were collected from two different workshops (workshop H and workshop L) of a sauce-flavored liquor factory in Erlang Town (Luzhou City, Sichuan Province, China). The two workshops are 10 km apart, both located at an altitude of approximately 300 m, at a geographical location of 28 degrees north latitude and 106 degrees east longitude. The fourth round of stacked fermentation typically lasts approximately 5 days, with samples obtained daily from the stacked piles. Samples were systematically collected from stratified layers (upper, middle, and lower strata) of the fermentation pile at multiple spatial locations to ensure representative sampling throughout the vertical profile of the stacked fermentation system. These samples from individual days were pooled and stored at −80 °C for amplicon sequencing and physicochemical factor analysis.

### 2.2. Amplicon Sequencing

Total DNA was extracted from the fermented grains using the Soil DNA Isolation Kit (FOREGENE, Chengdu, China), following the manufacturer’s instructions. For bacterial analysis, the V3-V4 region of the 16S rRNA bacterial gene was amplified using the universal primers 338F (5=-ACTCCTACGGGAGGCAGCAG-3=) and 806R (5=-GACTACHVGGGTWTCTAAT-3=). For fungi, the ITS2 region was amplified using the primers ITS2 (5=-GCTGCGTTCTTCATCGATGC-3=) and ITS3 (5=-GCATCGATGAAGAACGCAGC-3=). Purified PCR products were sequenced on an Illumina Hiseq 2500 platform at a biotechnology company. Sequencing adhered to standard protocols provided by Novogene Technology Co., Ltd. (Tianjin, China). Raw sequencing reads were demultiplexed, quality-filtered by fastp version 0.23.1 [16], and merged by FLASH version 1.2.11 [17]. Clean data were classified into operational taxonomic units (OTUs) with at least 97% similarity using UPARSE version 7.1. The Vsearch cluster OTU function was employed to generate the representative sequences [18]. Taxonomy annotation of each OTU was performed using RDP Classifier version 2.2 against the 16S rRNA and ITS databases [19].

### 2.3. Measurement of Physicochemical Indicators

The physicochemical properties of the fermented samples included the contents of starch, glucose, alcohol, acidity, titratable acidity, moisture, and temperature. Total starch content was determined using Folin’s reagent, following the procedures outlined by Chen et al. [20]. Glucose was detected using ultrahigh-performance liquid chromatography (UPLC, ACQUITY UPLC BEH C18 [100 mm × 2.1 mm, 1.7 μm]), Waters Corporation (Milford, MA, USA), equipped with an evaporative light-scattering detector. The mobile phase is 5 mmol/L H_2_SO_4_, the elution method is isocratic, the flow rate is 0.8 mL/min, the injection volume is 20 μL, the column temperature is 65 °C [21], and the concentrations of the glucose standard samples used in the experiment were 10 g/L, 5 g/L, 2.5 g/L, 1.25 g/L, 0.625 g/L, 0.3125 g/L, and 0.15625 g/L, respectively. Acidity was determined by titration with NaOH (0.1 M), using phenolphthalein as an indicator [22]. The moisture was determined gravimetrically by drying samples at 105 °C to a constant weight over at least 3 h. The daily temperature during fermentation was monitored in real-time at the sampling location. Ethanol and lactic acid content were quantified using high-performance liquid chromatography (HPLC) on a Waters 2695 system equipped with a Sunfire C18 column (150 mm × 4.6 mm, 5 μm) and a photodiode array detector. The column temperature is maintained at 30 °C, with a mobile phase of 5 mmol/L H_2_SO_4_, a flow rate of 0.3 mL/min, and an injection volume of 10 μL. The ethanol standard solutions were prepared at seven concentration levels: 1 g/L, 0.5 g/L, 0.25 g/L, 0.125 g/L, 0.0625 g/L, 0.03125 g/L, and 0.015625 g/L. A series of lactic acid standard samples were prepared with concentrations ranging from 2 g/L to 0.03125 g/L, including 2g/L, 1 g/L, 0.5 g/L, 0.25 g/L, 0.125 g/L, 0.0625 g/L, and 0.03125 g/L. The concentrations of ethanol and lactic acid are calculated based on the peak areas of the standard samples [21].

### 2.4. Analysis of Volatiles

Five grams of a sample and 20 mL of deionized water were placed into a 50 mL centrifuge tube and, after sufficient shaking, the sample was ultrasonicated for 30 min, and then centrifuged at 4 °C for 10 min at 8000× *g*. Then, 8 mL supernatant and 20 μL internal standard (2-Methyl-2-butanol, 100 μg/mL) was placed in a 20 mL headspace vial containing 3.0 g NaCl, tightly capped with a silicon septum, extracted with PDMS/CWR/DVB, equilibrated at 65 °C in a thermostatic bath for 15 min, and extracted for 20 min at the same temperature under stirring with a 400 r/min shaking speed. After extraction, the fiber was immediately introduced into the injection port of gas chromatography–mass spectrometry to thermally desorb the analyte at 250 °C for 180 s.

The volatiles were analyzed and identified on a Trace GC Ultra gas chromatograph-DSQ II mass spectrometer (Thermo Electron Corp., Waltham, MA, USA) equipped with an HP-INNOWAX capillary column (60.0 m × 0.25 mm × 0.25 μm, Agilent Technology, Santa Clara, CA, USA). Mass spectrum was generated in the electron impact (EI) mode at 70 eV ionization energy using the full scan mode (35 to 400 amu).

The following is the GC operation condition: the carrier gas was helium (purity > 99.999%) at a flow rate of 0.8 mL/min and the inlet temperature was 280 °C with splitless injection. The oven temperature was maintained at 35 °C for 1 min, followed by an increase of 3 °C/min to 280 °C, and then programmed to 230 °C at 6 °C/min, and held for 5 min. The flavor compounds were tentatively identified by matching mass spectrum with NIST05 spectrum database and verified by comparison of their Kováts retention indices (RI) with the RIs reported in other studies, which was calculated by using C8-C20 n-alkanes [23].

Quantitative analysis of flavor compounds was carried out using the internal standard method. In this process, the area of each chromatographic peak was automatically integrated, and the ratio of the peak area of the internal standard to the peak area of each component in the sample was calculated. As a result, the content of each flavor component was quantified.

### 2.5. Statistical Analysis

All assays were performed in triplicate, with each replicate analyzed in duplicate, and the results averaged. Diversity indices, including Chao1, Shannon, Simpson, ACE, and Goods-coverage, were calculated using Qiime software (Version 1.9.1). Correlations between physicochemical properties and microorganisms at the genus level were analyzed using distance-based redundancy analysis and Spearman correlation analysis in R (version 3.2.4). The data were subjected to Duncan’s multiple range test, with a significance level set at *p* < 0.05. Orthogonal Partial Least Squares Discriminant Analysis (OPLS-DA) was employed to develop a model that distinguishes between samples and volatile components, while Principal Component Analysis (PCA) was used to cluster samples based on the relative abundance of volatile components, with both processes being conducted using SIMCA 14.1. All the significantly correlated fungal and bacterial genera and volatiles were studied using the pheatmap software package software (Version 1.0.12) and visualized by heatmap graphs [24].

## 3. Results

### 3.1. Dynamic Changes in Physicochemical Factors

Throughout the stacked fermentation process, physicochemical factors—including moisture content, total acidity, starch content, glucose content, lactic acid concentration, ethanol concentration, and temperature—exhibited dynamic alterations. These changes influence microbial metabolic activities, which in turn shape the production of flavor compounds [25,26].

**Figure 1:**
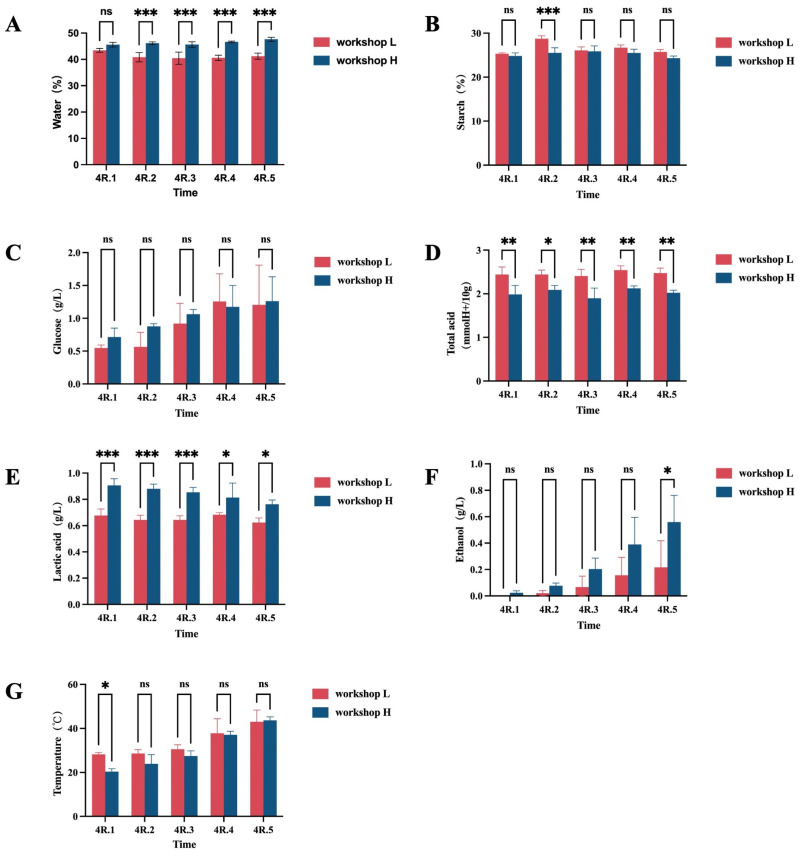
Dynamics of physicochemical characteristics. (**A**) Changes in moisture content. (**B**) Changes in starch content. (**C**) Changes in sugar content. (**D**) Changes in acidity content. (**E**) Changes in lactic acid content. (**F**) Changes in ethanol content. (**G**) Changes in Temperature. * represents significant difference between H and L. “ns” represents that there is no significant difference between the two groups; “*” indicates that there is a statistically significant difference between the two groups; “**” indicates that there is a significant difference between the two; “***” indicates that there is an extremely significant difference between the two groups.

Moisture content is a critical parameter in Baijiu production, significantly influencing microbial succession [27]. As illustrated in Figure 1A, the moisture content of the stacked fermented grains in workshop H ranged from 45% to 48%. No significant changes were observed during the first three days, followed by an increase on the fourth day. Conversely, the moisture content in workshop L ranged from 40% to 44%, and exhibited a decreasing trend during the first three days, with a subsequent increase on the fourth day. This difference may be attributed to the high evaporation rate in workshop L due to elevated fermentation temperatures during the early stage, followed by increased microbial activity and starch degradation in later stages, which contributed to higher moisture retention.

Starch, the primary energy source for microbial growth during Baijiu brewing, directly correlates with Baijiu yield. Starch content exhibited an increasing trend initially, followed by a decrease in both workshops (Figure 1B). Workshop L showed higher starch levels compared to workshop H, suggesting a lower abundance of amylase-secreting microorganisms in workshop L. This indicates that the starch consumption and utilization rates were less efficient in workshop L, highlighting the limited influence of stacking fermentation on starch content.

Glucose content reflects the balance between saccharification and fermentation rates. In both workshops, glucose content increased significantly during the fourth round of stacked fermentation, rising from 0.71% to 1.26% in workshop H and from 0.55% to 1.2% in workshop L, respectively (Figure 1C). This upward trend indicates the vigorous microbial decomposition activity and accelerated conversion of raw materials into glucose, outpacing their utilization rates.

Acidity modulates fungal structure and diversity and influences the production of volatile metabolites during Baijiu fermentation [28]. As depicted in Figure 1D, total acid remained relatively stable throughout the fermentation process, reflecting a dynamic equilibrium. This stability likely results from ongoing microbial metabolism, which produces organic acids while contributing to ester formation [29]. Nonetheless, the total acid in workshop H was consistently lower than in workshop L.

Lactic acid, a predominant acidic substances in sauce-flavor Baijiu, plays a crucial role in fermentation [30]. As shown in Figure 1E, lactic acid concentration declined in both workshops, with higher levels observed in workshop H. The declining trend is attributed to elevated stacked fermentation temperatures, which attenuated the activity of acid-producing strains. Additionally, lactic acid was consumed for ester synthesis, contributing to its depletion.

Ethanol was identified as a key factor governing microbial succession during fermentation, shaping Baijiu’s flavor profile [31]. Ethanol levels increased significantly in both workshops throughout the stacked fermentation process, with the most prominent rise observed in workshop H (Figure 1F).

Temperature is a paramount indicator of stacked fermentation efficiency [32]. During the initial stages, the temperature in workshop H was lower than in workshop L. However, as fermentation progressed, the temperature in workshop H increased more rapidly (Figure 1G). It was discovered that during the stacking fermentation process, the temperature exhibited a significant positive correlation with the yeast count. As the stacking time extended, the number of yeasts increased substantially. The metabolic heat generated by these microorganisms caused an elevation in the temperature of the fermented grains. This temperature increase, in turn, was conducive to the growth and reproduction of microorganisms [33]. It was hypothesized that the stacking fermentation in workshop H had a higher yeast count and more vigorous metabolic activities.

The light blue line represents workshop L, while the dark blue line represents workshop H.

The *X*-axis represents 4R.1, 4R.2, 4R.3, 4R.4, and 4R.5, which represent the first day to the fifth day of the fourth round of stacked fermentation.

### 3.2. Microbial Diversity and Community Composition During Stacked Fermentation

Table 1 summarizes the alpha diversity indices of the bacterial and fungal communities across different stages of the fourth round of stacked fermentation in workshop L (L.4R.1 to L.4R.5) and workshop H (H.4R.1 to H.4R.5).

For fungi, the Shannon and Simpson indices exhibited parallel trends, initially increasing and subsequently decreasing in both workshops, indicating greater fungal diversity during the middle and later stages of stacked fermentation. The Chao1 index exhibited continuous fluctuations throughout stacked fermentation, with a general increasing trend observed in workshop H and a decreasing trend in workshop L, reflecting divergent changes in fungal community structure between workshops. A total of 5,187,042 original sequences were obtained, including 1,273,411 bacterial sequences and 3,913,631 fungal sequences. After quality filtering, 4,556,138 valid sequences were retained, comprising 1,019,142 bacterial sequences and 3,536,996 fungal sequences. These sequences were clustered into 951 bacterial OTUs and 1120 fungal OTUs at a 97% similarity threshold.

As shown in Table 1, the sequencing coverage for both bacterial and fungal communities indicated sufficient sequencing depth and an accurate representation of microbial diversity in the fermented grains. For fungal communities, the Shannon and Simpson indices exhibited an increasing trend during the middle stages of fermentation, followed by a decline, indicating greater fungal diversity during these stages. The Chao1 index fluctuated but showed an overall increase in workshop H, reflecting more dynamic fungal community changes compared to the declining trend observed in workshop L.

For bacterial communities, the Shannon and Simpson indices showed a similar decreasing-then-increasing pattern in both workshops. In workshop H, bacterial diversity began to recover on the fourth day, while in workshop L, this recovery was observed on the fifth day. The lowest bacterial diversity was recorded on the third day in workshop H and on the fourth day in workshop L, reflecting different successional dynamics between the workshops.

### 3.3. Bacterial Community Succession During Stacked Fermentation

**Figure 2:**
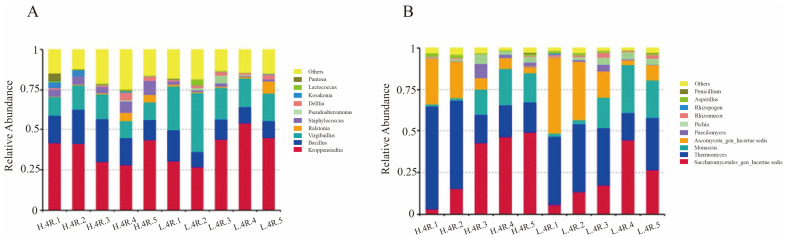
The succession of dominant microbial community. (**A**) Bacteria and (**B**) fungi. H.4R.1, H.4R.2, H.4R.3, H.4R.4, and H.4R.5 represent the first day to the fifth day of the fourth round of stacked fermentation in workshop H; L.4R.1, L.4R.2, L.4R.3, L.4R.4, and L.4R.5 represent the first day to the fifth day of the fourth round of stacked fermentation in workshop L. The others represent the genera with average relative abundance < 1%.

Following clustering, the bacterial communities were classified into 22 phyla, 41 orders, 98 families, and 167 genera. Among these, *Firmicutes* emerged as the dominant phylum, with relative abundances ranging from 70.3% to 89.6%. Genera with an average relative abundance exceeding 1% were categorized as dominant microorganisms, while those exceeding 10% were designated as absolutely dominant microorganisms [34]. The fluctuations in bacterial community composition during stacked fermentation are depicted in Figure 2A.

Notably, *Kroppenstedtia*, *Bacillus*, and *Virgibacillus* were identified as the unequivocal dominant bacterial genera in both workshops, which is consistent with their reported roles in high-temperature Daqu.

*Kroppenstedtia*, known for its involvement in color development and biosynthesis of organic acids [35], displayed distinct trends in the two workshops. In workshop H, *Kroppenstedtia* abundance decreased during the first four days and peaked on the fifth day, achieving absolute dominance. In contrast, *Kroppenstedtia* in workshop L increased from the third day, peaked on the fourth day, and declined slightly on the fifth day. *Bacillus*, renowned for its production of amylase and proteases, plays a pivotal role in liquefaction and saccharification [36]. In workshop H, *Bacillus* abundance increased during the early stages and peaked at 26.4% on the third day, followed by a decline. However, its abundance in workshop L was significantly lower throughout fermentation, likely due to differences in workshop environmental conditions.

*Virgibacillus* exhibited an increase followed by a decrease in abundance. The highest abundance of *Virgibacillus* was recorded on the second day in workshop L and the third day in workshop H. Workshop L consistently showed higher *Virgibacillus* abundance (*p* < 0.05), positioning it as the second most dominant genus in workshop L.

Furthermore, *Ralstonia* showed an increase in abundance toward the end of the fermentation period, suggesting its potential involvement in the later stages of microbial succession. Other genera, such as *Staphylococcus* and *Pseudoalteromonas*, exhibited dynamic fluctuations. *Staphylococcus* showed slightly higher abundance in workshop H, while *Pseudoalteromonas* peaked at 4.7% on the third day but remained below 1% at other stages. These genera, consistent with findings from other Baijiu production areas [37], may contribute to flavor compound formation.

### 3.4. Fungal Community Succession During Stacked Fermentation

Fungal community analysis revealed a total of 11 phyla, 36 orders, 78 families, 160 genera, and 378 species. *Ascomycota* emerged as the predominant fungal phylum, comprising 96.3% to 99.4% of the relative abundance across samples. The absolute dominant genera included *Saccharomycetales, Thermomyces, Monascus,* and *Ascomycota*, with dynamic changes over the stacked fermentation process (Figure 2B), and are known for the ethanol production and enhancing flavor diversity [38]_._ *Saccharomycetales* abundance occurred from day 1 to day 4, slowing on day 5. Its abundance rose from 5.5% to 26.9% in workshop L and from 3.2% to 48.9% in workshop H. Moreover, its abundance reaches the highest in workshop H on the 5th day, while in workshop L, its abundance reaches the maximum on the 4th day. And its abundance in workshop H is significantly lower than that in workshop L.

This *thermophilic* genus, integral to saccharification and flavor development, declined over the fermentation period. In workshop H, its abundance dropped from 61.5% to 18.3%, while in workshop L, it declined from 41.2% to 31.2%, with a slight recovery on day 5. The decreasing trend is more obvious in workshop H, which corresponds to the obvious increasing trend of *Saccharomycetales* in workshop H. Initially scarce, *Monascus* abundance increased significantly by day 3, peaking on day 4. It rose from 1.4% to 15.5% in workshop H and from 1.6% to 18.7% in workshop L. This genus plays a vital role in saccharification and esterification, producing enzymes like protease and amylase, which are essential for ethanol and ester production [37]. *Ascomycota* displayed a decreasing trend, particularly in workshop L, where its abundance dropped from 45.8% on day 1 to 8.6% on day 5. In workshop H, a milder decline was observed, suggesting differences in environmental conditions between workshops.

*Paecilomyces* and *Pichia* exhibited minimal abundance during the first two days but increased rapidly by day 3, reaching 3.7% and 4%, respectively. Aspergillus showed an opposing trend, with its abundance declining from >1% on days 1–2 to <1% by day 3.

The dynamic shifts in fungal abundance highlight the significant impact of physicochemical changes during fermentation, particularly on day 3, when fluctuations in temperature, ethanol, and glucose levels had the most pronounced influence on fungal growth.

### 3.5. Relationships Between Physicochemical Factors and Microbial Communities

**Figure 3:**
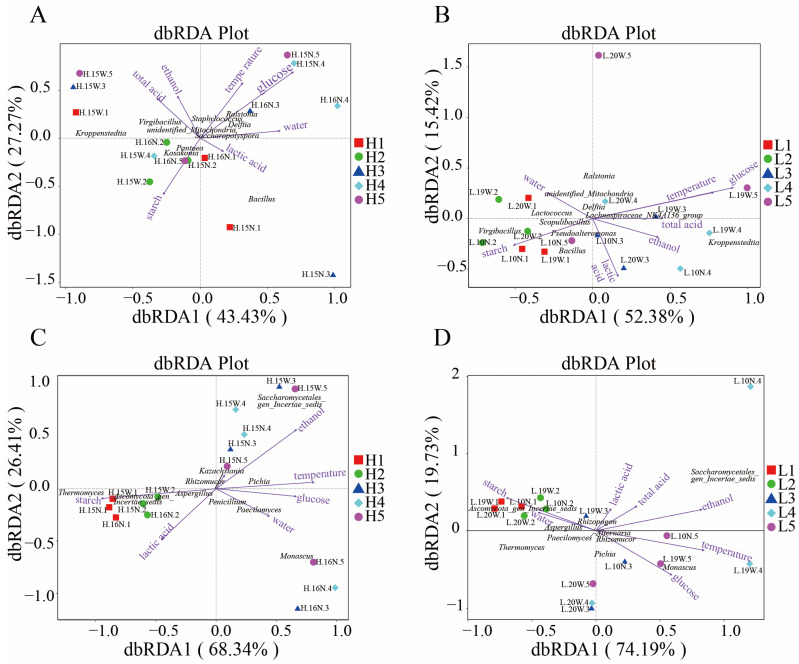
db-RDA between physicochemical factors and dominant bacteria genera in workshops H (**A**) and L (**B**). Physicochemical factors and dominant fungal genera in workshops H (**C**) and L (**D**).

**Figure 4:**
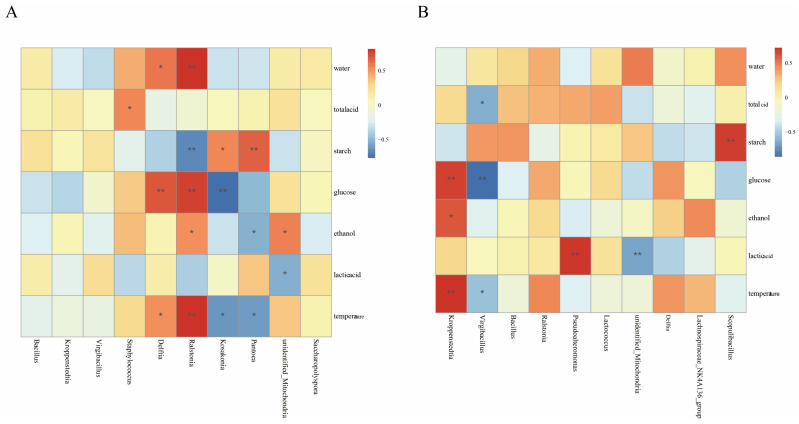
Spearman′s correlation analysis between physicochemical factors and dominant bacteria genera in workshops H (**A**) and L (**B**). “*” indicates a significant correlation (*p* < 0.05), “**” indicates an extremely significant correlation (*p* < 0.01).

**Figure 5:**
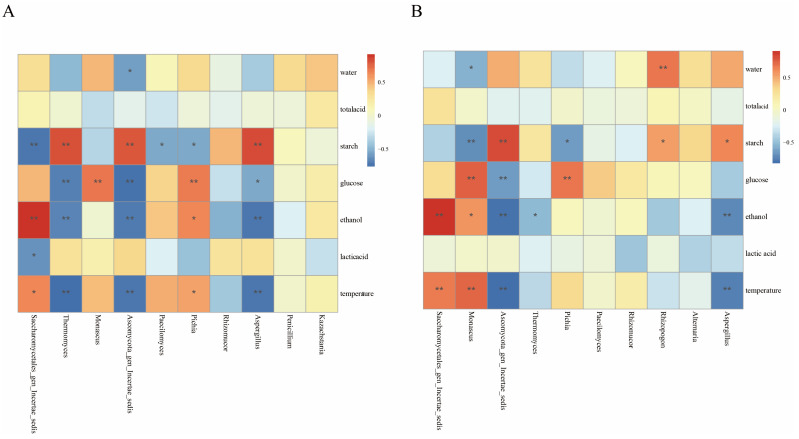
Spearman′s correlation analysis between physicochemical factors and dominant fungal genera in workshops H (**A**) and L (**B**). “*” indicates a significant correlation (*p* < 0.05), “**” indicates an extremely significant correlation (*p* < 0.01).

Variations in physicochemical factors, such as ethanol, moisture, lactic acid, glucose, starch, and temperature, during fermentation significantly influence microbial community [39]. To elucidate these relationships, distance-based redundancy analysis (db-RDA) and Spearman’s correlation analysis were conducted (Figure 3, Figure 4 and Figure 5). The db-RDA results unveiled that the physicochemical index explains 70.7% of the bacterial community variance in workshop H and 67.8% in workshop L. In workshop H, genera such as *Delftia* and *Ralstonia* positively correlated with moisture, glucose, ethanol, and temperature (*p* < 0.01). Conversely, in workshop L, *Virgibacillus* abundance negatively correlated with acid, glucose, and temperature (*p* < 0.01). Physicochemical factors accounted for 94.75% and 93.92% of fungal community variance in workshops H and L, respectively. *Saccharomycetales* exhibited significant positive correlations with temperature and ethanol, particularly in workshop H. In contrast, *Ascomycota_gen_Incertae_sed*, *Thermomyces*, and *Aspergillus* showed a negative correlation with glucose, ethanol, and temperature and a positive correlation with starch in workshop H; however, in workshop L, *Ascomycota_gen_Incertae_sed* has the same correlation as in workshop H, and *Aspergillus* has the same correlation with ethanol, temperature, and starch as in workshop H, but only *Thermomyces* has a negative correlation with ethanol. Glucose, starch, ethanol, and temperature emerged as the primary drivers of microbial succession during stacked fermentation. For example, *Saccharomycetales* positively correlated with ethanol and temperature, which progressively increased, fostering its dominance in workshop H. *Monascus* and *Pichia* exhibited positive correlations with glucose and negative correlations with starch, underscoring starch being broken down into glucose and the role of glucose availability in promoting fungal growth and metabolic activity. *Thermomyces* abundance was negatively influenced by ethanol, highlighting its sensitivity to rising ethanol levels. These findings underscore how variations in endogenous factors between workshops influence microbial succession and community composition.

### 3.6. Relationships Between Flavor Substances and Microbial Communities

**Figure 6:**
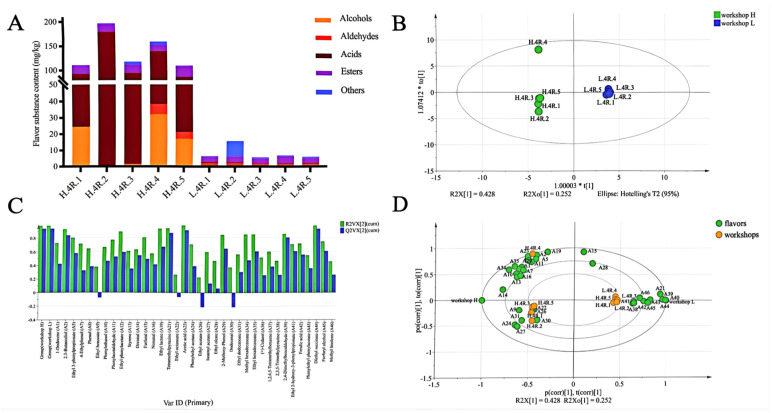
Flavor substance analysis in workshops H and L. (**A**) Changes in flavor substance content in two workshops; (**B**) OPLS-DA score of flavor substances in different workshop brewing processes; (**C**) PCA of flavor substances in different workshops; (**D**) score plot of OPLS-DA for different flavor substances.

During the stacking process, there were significant differences in the types and contents of flavor substances between the two workshops. A total of 46 flavor substances were detected in both workshops, with 35 detected in workshop H and 23 in workshop L (Table S1). The flavor substances were primarily composed of esters, alcohols, acids, and other compounds (Figure 6A). In workshop H, the higher-content flavor substances included acetic acid, ethyl palmitate, phenylethyl alcohol, and phenylethyl acetate, while workshop L was dominated by 2,4-dimethylbenzaldehyde, phenylethyl phenylacetate, and tetramethylpyrazine. Notably, the total content of flavor substances in workshop H was significantly higher than that in workshop L (Figure 6A).

OPLS analysis revealed significant differences in flavor substances between the two workshops (Figure 6B). Figure 6C illustrates the differences in the content of flavor substances between the workshops, while Figure 6D shows the distribution of different types of flavor substances in the two workshops. Notably, phenylethyl alcohol, the most important alcohol in Baijiu, showed a significant difference in content between the two workshops (Figure 6C). At the end of the stacking process, the content of phenylethyl alcohol in workshop H reached 16.464 mg/kg (Table S1), while it was not detected in workshop L. Furfural, a representative aldehyde in sauce-flavor Baijiu, was consistently higher in workshop L than in workshop H at all stages of the stacking process (Figure 6C). Acetic acid, a precursor for the synthesis of ester compounds, exhibited an initial increase followed by a decrease in workshop H during the stacking fermentation process. On the third day of stacking, the acetic acid content in workshop H was 390 times higher than that in workshop L (Table S1). Esters were the most abundant type of flavor substances in both workshops, with ethyl palmitate being the dominant compound. The contents of ethyl acetate and phenylethyl acetate in workshop H were significantly higher than those in workshop L. In contrast, the contents of ethyl phenylacetate, ethyl lactate, and ethyl oleate in workshop L showed a notable increase on the fourth and fifth days of stacking fermentation (Figure 6D).

**Figure 7:**
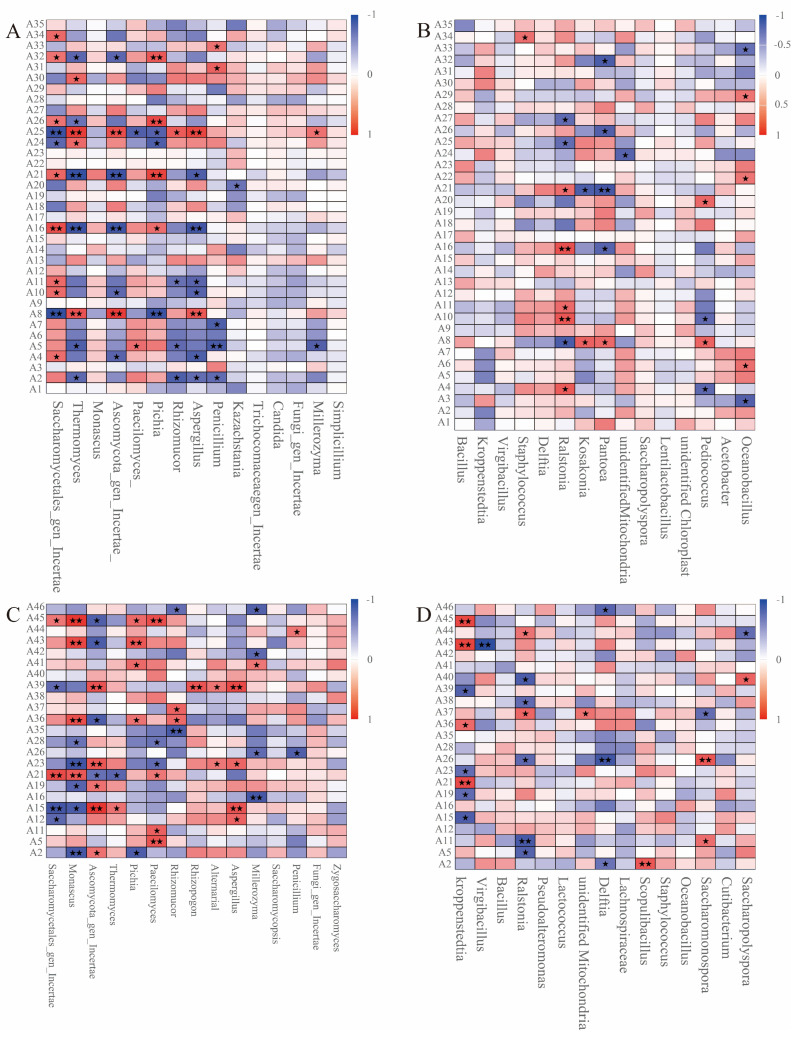
Spearman correlation of flavor substances and microbial communities. (**A**) Relationship between flavor substances and fungal community in workshop H; (**B**) relationship between flavor substances and bacterial community in workshop H; (**C**) relationship between flavor substances and fungal community in workshop L; (**D**) relationship between flavor substances and bacterial community in workshop L. “*” indicates a significant correlation (*p* < 0.05), “**” indicates an extremely significant correlation (*p* < 0.01).

Spearman correlation analysis revealed the relationships between microorganisms and flavor substances in the different workshops (Figure 7). In workshop H, dominant fungi such as *Saccharomycetales*, *Thermomyces*, *Ascomycota*, and *Pichia* showed significant positive correlations with multiple flavor substances. Notably, *Saccharomycetales* was significantly positively correlated with eight flavor substances, including ethyl acetate. Although *Thermomyces* exhibited significant negative correlations with six flavor substances, it was positively correlated with four others, such as phenylethyl acetate (Figure 7A). These results validate the higher contents of ethyl acetate and phenylethyl acetate in workshop H (Figure 6D). The dominant bacteria in workshop H showed fewer correlations with flavor substances, but *Ralstonia* and *Oceanobacillus* were significantly positively correlated with five and three flavor substances, respectively (Figure 7B). In workshop L, fungi such as *Monascus*, *Ascomycota*, *Pichia*, *Paecilomyces*, and *Aspergillus* were significantly positively correlated with multiple flavor substances, particularly *Ascomycota*, which showed significant positive correlations with five flavor substances (Figure 7C). However, *Saccharomycetales* with the highest abundance in the L workshop was only positively correlated with furfuryl alcohol and tetramethylpyrazine and showed no strong correlation with other flavor substances. *Kroppenstedtia* with the highest abundance in workshop L was significantly positively correlated with furfuryl alcohol, cedryl alcohol, phenethyl phenylacetate, and tetrmethylpyrazine, while *Ralstonia* was also significantly positively correlated with the two flavor substances (Figure 7D).

## 4. Discussion

The production of sauce-flavor Baijiu is greatly influenced by the environment, and there are differences in the quality of the liquor produced in different workshops. The fourth round is the core round in the production of sauce-flavor Baijiu, and it has a very strong representativeness because of its high ethanol production rate and good Baijiu quality 14. According to current research, it is still unclear what differences exist in the fermentation process of different workshops in Erlang Town. In our study, there are differences in the succession patterns of dominant microbial genera between workshop H and workshop L, and the physicochemical factors also have different driving effects on the microorganisms.

### 4.1. Differences in Key Microorganisms Between Two Workshops

The succession of microorganisms in sauce-flavor Baijiu accounted for the differences between the different workshops [2]. Thus, focusing on the succession pattern of the microbial community during fermentation can more effectively clarify these disparities. Firstly, the root cause of this variation lies in the existence of core microorganisms in the fermentation process [40]. Although the community composition of microorganisms is similar, the succession patterns vary.

In our study, the results indicated that *Kroppenstedtia*, *Bacillus*, and *Virgibacillus* were identified as the dominant bacterial genera in both workshops. However, their abundances and trends diverged (as shown in Figure 2). These genera played a crucial part in the succession of bacterial communities, and this finding was consistent with previously reported results [14].

Notably, *Kroppenstedtia* was the bacterial genus with the highest abundance in both workshops. However, there were significant differences in the second most dominant bacterium. In workshop H, it was *Bacillus*, while in workshop L, it was *Virgibacillus*. Moreover, the three dominant bacterial genera showed distinctly different changing trends during the stacked fermentation process. This might be because, as the fermentation time prolonged, the physicochemical properties of the fermented grains continuously changed, which further influenced the growth and metabolism of various dominant bacteria. At the same time, multiple microorganisms competed with each other, causing the community structure to keep changing [22].

On the other hand, the fungal community also had a profound impact on fermentation. In the fourth round of stacked fermentation, the dominant fungal genus with the highest abundances were similar, including *Saccharomycetales*, *Thermomyces*, *Monascus*, and *Ascomycota_gen_Incertae_sed*. Moreover, the succession patterns were also similar. However, there were significant differences in the abundances of each genus. The top two dominant microorganisms, *Saccharomycetales* and *Thermomyces*, showed opposite succession trends in the two workshops as the fermentation time extended. There may be a competitive relationship between these two genera [41]. The abundance of *Monascus* reached its peak on the fourth day of fermentation in both workshops, indicating that the environment in the later stage of fermentation is more conducive to its growth. As the fermentation time lengthened, the abundance of *Ascomycota_gen_Incertae_sed* showed a downward trend in both workshops, yet its abundance in workshop L was significantly higher than that in workshop H. This suggests that the initial stacked fermentation conditions of the grains in workshop L might be more favorable for the growth of *Ascomycota_gen_Incertae_sed*. The dynamic changes in microorganisms in the two workshops demonstrate that, for the same fermentation round, there are commonalities in the dominant genera among different production workshops, but differences exist in their succession patterns.

### 4.2. Influence Mechanism of Physicochemical Factors on Microorganisms

The changes in the community were mainly attributed to the variations in relevant physicochemical parameters during the stacked fermentation process [25]. According to the results of the redundancy analysis (RDA), glucose, starch, ethanol, and temperature were identified as the key factors driving the succession of microbial communities. As the fermentation time extended, the dynamic change trends of the physicochemical parameters of the fermented grains in the two workshops were basically consistent. The overall content of glucose showed an upward trend, while that of starch showed a downward trend. Correlation analysis indicated that *Ascomycota* was significantly positively correlated with starch and significantly negatively correlated with glucose. Moreover, during the stacked fermentation process, the succession pattern of *Ascomycota* was the same as the change pattern of starch. The abundance of *Ascomycota_gen_Incertae_sed* and the content of starch in workshop L were significantly higher than those in workshop H. This suggests that during the stacked fermentation process, the amylase and glucoamylase produced by *Ascomycota_gen_Incertae_sed* play an important role in the conversion of starch into glucose and meanwhile provide sufficient energy for subsequent alcohol fermentation [42].

However, the driving effects of these physicochemical factors on microorganisms varied between the two workshops. In workshop H, *Saccharomycetales* and *Thermomyces* were significantly correlated with starch, while in workshop L, *Monascus* was significantly negatively correlated with starch. This may be related to the differences in the starch content of the fermented grains under the initial conditions of the two workshops, which drive the growth of different microorganisms. Meanwhile, we found that the change in glucose content was consistent with the succession pattern of *Saccharomycetales*, indicating that there is a certain relationship between the rate of starch conversion into glucose and the growth and metabolism of *Saccharomycetales* [37]. Among the major bacterial genera, *Kroppenstedtia* in workshop L was significantly positively correlated with glucose. This may be because the fermented grains at the initial fermentation stage in workshop L contained more starch, which was more conducive to driving *Kroppenstedtia* to produce some enzymes that act on the polysaccharides in the fermented grains and gradually decompose them into glucose [43]. Meanwhile, we also found that although there were significant differences in the contents of glucose and starch between the two workshops at the beginning of fermentation, the differences were minimal at the end of stacked fermentation. This might be the result of the joint metabolism of multiple microorganisms, indicating that after the cooperative fermentation by multiple microorganisms, the differences in glucose and starch between the two workshops have decreased, respectively.

Also worth our attention was that both temperature and ethanol showed an upward trend during the stacked fermentation process. They were both significantly negatively correlated with *Ascomycota* and *Aspergillus* and positively correlated with *Saccharomycetales* and *Kroppenstedtia*. Moreover, the abundance of *Saccharomycetales* increased during the fermentation process. This indicates that *Saccharomycetales* can convert the glucose produced by the metabolism of *Ascomycota_gen_Incertae_sed* and *Aspergillus* into alcohol [37]. The biothermal energy generated during its metabolism gradually raises the temperature of the fermented grains [33], making the fermentation conditions more and more suitable for its growth. These microorganisms work synergistically in the fermented grains to convert and utilize the starch in the raw materials, jointly promoting the progress of fermentation. Notably, although the temperatures of the fermented grains in the two workshops were different at the beginning of fermentation, the difference was negligible at the end of fermentation. However, ethanol showed the opposite trend. This suggests that there may be significant differences in the fermentation influence mechanisms of ethanol in different workshops. Workshop H, where *Saccharomycetales* has a higher abundance, has a more pronounced impact on ethanol.

Throughout the fermentation process, the total acid content remained relatively stable, indicating a state of dynamic equilibrium. Contrary to the total acid, the lactic acid content in workshop L was significantly lower than that in workshop H. We found that *Saccharomycetales* in workshop H was negatively correlated with lactic acid. This might be related to the consumption of lactic acid by *Saccharomycetales* for the formation of esters [37].

To sum up, the microbial composition structures in different workshops during the same fermentation round are similar. There are differences in the succession patterns and abundances of bacteria, while the succession patterns of fungi are relatively consistent. However, there are significant differences in the abundances of dominant fungi. The succession of the microbial community is driven by the initial environmental factors of the grains, especially by key microorganisms. Nevertheless, through the synergistic fermentation of microorganisms, the differences in these physicochemical factors in the fermented grains become smaller at the end of the stacked fermentation process.

### 4.3. Microbial-Mediated Flavor Formation

The differences in flavor substances between the two workshops may be related to the brewing processes and raw materials used [44]. For instance, the higher furfural content in workshop L could be associated with the amount or variety of rice husk added, which further influences the accumulation of aldehyde compounds [44]. Additionally, the significant accumulation of phenylethyl alcohol in workshop H and its absence in workshop L indicate distinct microbial metabolic activities between the two workshops [45]. These differences may stem from variations in fermentation conditions, microbial community composition, or process parameters, ultimately leading to significant disparities in the types and contents of flavor substances. These findings provide important insights for further optimizing brewing processes and regulating flavor profiles. It is noteworthy that the contents of ethyl acetate and phenylethyl acetate in the esters of workshop H are significantly higher than those in workshop L. This may be due to the high content of acetic acid in workshop H, which accelerates the esterification reaction [46]. In workshop L, the contents of ethyl phenylacetate, ethyl lactate, and ethyl oleate suddenly increased on the fourth and fifth days of the stacking fermentation process, indicating an increase in the rate of esterification toward the end of the process [47]. The accumulation of these flavor substances laid the foundation for the subsequent fermentation of sauce-flavor Baijiu, ultimately enhancing the aroma of the finished product.

The composition and metabolic activities of microbial communities play a crucial role in the formation of flavor substances. In workshop H, the positive correlations between fungi such as *Saccharomycetales* and *Thermomyces* and various flavor substances, particularly the significant associations with ethyl acetate and phenylethyl acetate, are likely the main reasons for the higher content of these ester compounds in workshop H. In contrast, the weaker correlations between *Saccharomycetales* and flavor substances in workshop L may be attributed to the lower content of precursors such as acetic acid, which inhibits the esterification process [48]. Additionally, the positive correlations between microorganisms like *Ascomycota* and *Kroppenstedtia* and multiple flavor substances in workshop L indicate that these microorganisms play an important role in flavor formation in workshop L. Notably, *Ralstonia* showed positive correlations with various flavor substances in both workshops, which may be related to its ability to produce amylase and promote the conversion of glucose into acids, thereby indirectly influencing the synthesis of flavor substances [49]. These findings provide important insights for further elucidating the relationship between microorganisms and flavor substances, as well as theoretical support for optimizing brewing processes to regulate the formation of specific flavor compounds.

## 5. Conclusions

In conclusion, this study clearly demonstrates that physicochemical factors in different workshops have a significant impact on microorganisms in fermented grains and the flavor. The succession of the microbial community was propelled by the initial environmental factors inherent in the fermented grains, including glucose, starch, ethanol concentration, and temperature. Notably, the functions of key microorganisms including *Saccharomycetales*, *Thermomyces*, *Monascus*, *Ascomycota_gen_Incertae_sed, Kroppenstedtia*, *Bacillus*, and *Virgibacillus* were of critical significance. These alterations in the microbial makeup, in return, exerted a profound impact on the production of flavor-related metabolites within the fermented grains. For future research in this domain, it is advisable to concentrate on a comprehensive exploration of the specific metabolic pathways of microorganisms under diverse physicochemical conditions. Additionally, efforts should be directed toward the formulation of more refined control strategies to optimize the liquor-making process.

## Figures and Tables

**Table 1:** α-Diversity indexes of bacterial and fungal communities during different times of fourth round of stacked fermentation in workshop L and workshop H.

Samples	Shannon		Simpson		Chao1		Ace		Goods_Coverage	
	Bacteria	Fungi	Bacteria	Fungi	Bacteria	Fungi	Bacteria	Fungi	Bacteria	Fungi
L.4R.1	3.642	2	0.85	0.629	134.071	177.75	135.01	179.598	0.9999	0.9999
L.4R.2	3.614	2.424	0.816	0.696	131.765	170.85	132.527	174.381	0.9999	0.9999
L.4R.3	3.526	2.838	0.838	0.786	106.248	171.13	105.793	173.517	0.9998	0.9997
L.4R.4	2.996	2.621	0.796	0.72	124.012	176.23	119.945	183.195	0.9999	0.9999
L.4R.5	3.521	2.925	0.83	0.79	140.376	187.04	137.677	187.074	0.9999	0.9998
H.4R.1	3.647	1.807	0.844	0.529	142.999	217.863	143.95	220.22	0.9999	0.9999
H.4R.2	3.485	2.396	0.844	0.645	144.424	254.32	146.77	235.034	0.9997	0.9998
H.4R.3	3.401	2.908	0.791	0.777	124.437	222.557	123.038	230.22	0.9998	0.9999
H.4R.4	4.027	2.517	0.896	0.718	107.333	170.113	106.704	177.512	0.9998	0.9999
H.4R.5	3.548	2.855	0.846	0.76	83.032	234.322	83.607	234.205	0.9999	0.9999

Note: Samples for workshop L were collected from L.4R.1, L.4R.2, L.4R.3, L.4R.4, and L.4R.5, and samples for workshop H were collected from H.4R.1, H.4R.2, H.4R.3, H.4R.4, and H.4R.5.

## Data Availability

The original contributions presented in this study are included in the article/Supplementary Material. Further inquiries can be directed to the corresponding author.

## Supplementary Materials

The following supporting information can be downloaded at: https://www.mdpi.com/article/10.3390/foods14060924/s1, Table S1. Varieties and Concentrations of Flavor Compounds in Different Workshops (mg/kg).
