# Supplementary material for: Temporal and Workshop Heterogeneity of Microbial Communities with Physicochemical Properties and Flavor Substances During Stacked Fermentation of Sauce-Flavor Baijiu
Source: Foods. 2025 Mar 8;14(6):924. doi: 10.3390/foods14060924 (PMC11941515; doi:10.3390/foods14060924)
Supplement: Supplementary file 1 [file foods-14-00924-s001.zip › foods-3474430-supplementary.pdf]

Tables

Table S1. Varieties and Concentrations of Flavor Compounds in Different Workshops (mg/kg)

	number	H.4R.1	H.4R.2	H.4R.3	H.4R.4	H.4R.5	L.4R.1	L.4R.2	L.4R.3	L.4R.4	L.4R.5
1-Dodecene	A1	0.168±0.007	ND	0.063±0.02	0.254±0.048	ND	ND	ND	ND	ND	ND
2.3-Butanediol	A2	ND	ND	0.901±0.191	2.525±0.161	ND	0.078±0.019	0.064±0.005	0.062±0.023	0.161±0.023	0.027±0.001
2-Nonanol	A3	ND	ND	0.066±0.013	ND	0.111±0.01	ND	ND	ND	ND	ND
2-n-pentylfuran	A4	0.635±0.15	ND	ND	ND	1.146±0.131	ND	ND	ND	ND	ND
Ethyl 3-phenylpropionate	A5	ND	ND	ND	0.253±0.038	0.147±0.007	ND	0.016±0.002	0.021±0.001	0.016±0.001	ND
3-Methyl-1-butene	A6	ND	ND	2.362±0.307	0.052±0.009	ND	ND	ND	ND	ND	ND
4-Ethylphenol	A7	ND	1.573±0.113	2.146±0.022	3.756±1.252	ND	ND	ND	ND	ND	ND
Phenol	A8	ND	0.284±0.054	1.488±0.014	1.667±0.514	ND	ND	ND	ND	ND	ND
Ethyl benzoate	A9	1.473±0.11	0.492±0.069	0.366±0.08	0.348±0.097	ND	ND	ND	ND	ND	ND
Phenylethanol	A10	24.435±0.66	ND	ND	29.422±0.332	16.464±0.866	ND	ND	ND	ND	ND

Phenylacetaldehyde	A11	ND	ND	ND	5.188±0.975	3.473±0.244	ND	0.123±0.011	0.099±0.007	0.099±0.001	ND
Ethyl phenylacetate	A12	0.17±0.027	ND	0.17±0.014	0.681±0.136	0.405±0.1	0.097±0.008	0.089±0.001	0.083±0.023	0.07±0.003	0.091±0.026
Styrene	A13	ND	0.121±0.008	ND	0.16±0.038	0.082±0.017	ND	ND	ND	ND	ND
Decanal	A14	0.099±0.006	ND	0.047±0.01	0.074±0.013	0.081±0.019	ND	ND	ND	ND	ND
Furfural	A15	ND	0.082±0.003	ND	0.652±0.129	ND	0.265±0.015	0.205±0.047	0.163±0.044	0.109±0.007	0.18±0.011
Nonanal	A16	ND	ND	0.272±0.105	0.393±0.02	0.458±0.045	0.013±0.004	ND	ND	0.085±0.021	ND
Ethyl nonanoate	A17	ND	0.682±0.061	ND	0.196±0.017	ND	ND	ND	ND	ND	ND
Ethyl cinnamate	A18	0.034±0.002	ND	0.023±0.005	ND	ND	ND	ND	ND	ND	ND
Ethyl lactate	A19	0.217±0.027	ND	ND	2.561±0.141	ND	0.147±0.014	0.137±0.036	0.084±0.022	0.087±	0.137±0.023
Ethyl tetradecanoate	A20	0.18±0.012	ND	0.071±0.002	ND	ND	ND	ND	ND	ND	ND
Tetramethylpyrazine	A21	ND	ND	0.093±0.01	0.09±0.003	0.11±0.028	0.23±0.044	0.257±0.045	0.319±0.035	0.293±0.021	0.323±0.1
Ethyl octanoate	A22	ND	0.86±0.045	0.029±0.005	0.221±0.051	ND	ND	ND	ND	ND	ND
Acetic acid	A23	68.378±5.433	179.329±14.657	93.429±11.854	101.296±13.102	65.777±11.983	0.628±0.041	0.386±0.013	0.239±0.024	0.278±0.002	0.384±0.058

Phenylethyl acetate	A24	8.115±0.185	5.351±0.391	ND	ND	6.268±1.073	ND	ND	ND	ND	ND
Octyl acetate	A25	0.068±0.005	1.109±0.103	ND	ND	ND	ND	ND	ND	ND	ND
Ethyl acetate	A26	ND	ND	6.07±0.489	ND	10.189±0.812	0.08±0.012	0.062±0.016	0.049±0.012	0.056±0.001	0.047±0.01
Isoamyl acetate	A27	3.25±0.662	7.592±0.526	6.351±0.172	0.262±0.021	ND	ND	ND	ND	ND	ND
Ethyl oleate	A28	0.183±0.031	ND	ND	1.017±0.001	ND	0.202±0.044	0.115±0.012	0.075±0.005	0.137±0.003	0.091±0.025
2-Methoxy-Phenol	A29	ND	0.072±0.014	0.538±0.015	0.023±0.003	ND	ND	ND	ND	ND	ND
Dodecanal	A30	0.022±0.003	0.386±0.001	ND	ND	0.023±0.007	ND	ND	ND	ND	ND
Ethyl dodecanoate	A31	0.023±0.005	0.042±0.009	ND	ND	0.073±0.006	ND	ND	ND	ND	ND
n-Hexanol	A32	ND	ND	0.244±0.066	ND	0.376±0.032	ND	ND	ND	ND	ND
n-Octanaldehyde	A33	0.04±0.006	ND	ND	ND	0.04±0.001	ND	ND	ND	ND	ND
Methyl hexadecanoate	A34	ND	0.039±0.015	0.022±0.003	0.091±0.017	0.03±0.002	ND	ND	ND	ND	ND
Ethyl hexadecanoate	A35	4.992±1.048	ND	3.237±0.588	7.77±2.769	4.19±0.511	0.526±0.003	0.396±0.072	0.292±0.016	0.553±0.152	0.36±0.047
(+)-Cedarol	A36	ND	ND	ND	ND	ND	ND	ND	0.015±0.001	0.01±0.002	0.016±0.003

1,2,4,5-Tetramethylbenzene	A37	ND	ND	ND	ND	ND	0.02±0.005	ND	0.019±0.008	0.013±0.003	0.032±0.012
2,3,5-Trimethylpyrazine	A38	ND	ND	ND	ND	ND	ND	0.011±0.003	0.012±0.003	0.011±0.001	ND
2,4-Dimethylbenzaldehyde	A39	ND	ND	ND	ND	ND	0.495±0.025	0.47±0.054	0.302±0.027	0.314±0.042	0.426±0.091
2,4-Di-tert-butylphenol	A40	ND	ND	ND	ND	ND	ND	3.542±0.626	0.023±	ND	ND
Ethyl 2-hydroxy-3-phenylpropionate	A41	ND	ND	ND	ND	ND	ND	0.063±0.007	0.048±0.011	0.044±0.011	0.055±0.018
Ferulic acid	A42	ND	ND	ND	ND	ND	0.012±0.002	0.01±0.001	0.008±0.002	0.026±0.004	0.01±0.002
Phenylethyl phenylacetate	A43	ND	ND	ND	ND	ND	ND	ND	0.418±0.11	0.367±0.06	0.434±0.078
Diethyl succinate	A44	ND	ND	ND	ND	ND	0.018±0.001	0.018±0.001	0.016±0.004	0.019±0.004	0.021±0.001
Furfuryl alcohol	A45	ND	ND	ND	ND	ND	ND	0.015±0.004	0.023±0.003	0.02±0.006	0.02±0.002
Methyl linoleate	A46	ND	ND	ND	ND	ND	0.14±0.07	0.086±0.005	0.039±0.004	0.161±0.008	ND
